# Biomimetic mineralisation systems for in situ enamel restoration inspired by amelogenesis

**DOI:** 10.1007/s10856-021-06583-x

**Published:** 2021-08-28

**Authors:** Jue Wang, Zhihui Liu, Bingyu Ren, Qian Wang, Jia Wu, Nan Yang, Xin Sui, Lingfeng Li, Meihui Li, Xiao Zhang, Xinyue Li, Bowei Wang

**Affiliations:** 1 Department of Obsterics and Gynecology, The Second Hospital of Jilin University, Changchun, Jilin China; 2 Department of Prosthodontics, Hospital of Stomatology, Jilin University, Changchun, Jilin China; 3 Department of Thyroid surgery, The Second Hospital of Jilin University, Changchun, Jilin China

## Abstract

Caries and dental erosion are common oral diseases. Traditional treatments involve the mechanical removal of decay and filling but these methods are not suitable for cases involving large-scale enamel erosion, such as hypoplasia. To develop a noninvasive treatment, promoting remineralisation in the early stage of caries is of considerable clinical significance. Therefore, biomimetic mineralisation is an ideal approach for restoring enamel. Biomimetic mineralisation forms a new mineral layer that is tightly attached to the surface of the enamel. This review details the state-of-art achievements on the application of amelogenin and non-amelogenin, amorphous calcium phosphate, ions flow and other techniques in the biomimetic mineralisation of enamel. The ultimate goal of this review was to shed light on the requirements for enamel biomineralisation. Hence, herein, we summarise two strategies of biological minimisation systems for in situ enamel restoration inspired by amelogenesis that have been developed in recent years and compare their advantages and disadvantages.

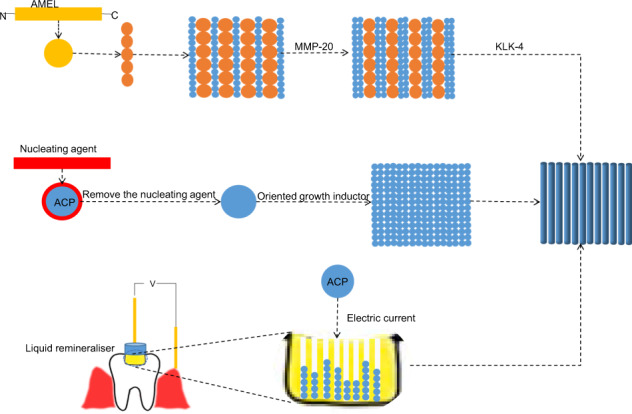

## Introduction

Dental decay and erosion are prevalent chronic diseases globally that cause massive mineral loss of enamel [1, 2]. Conventional enamel restoration strategies involve the mechanical removal of the decay and filling with artificial materials since enamel is acellular and cannot regenerate [3–5]. However, secondary caries frequently develop over time in the gap caused by the shrinkage between the restorative material and the tooth. Hence, in situ regrowth of enamel with a dense interface is an ideal strategy for enamel restoration.

Enamel, which is the hardest tissue in the human body, is composed of 96 wt.% inorganic material and 4 wt.% organic material and water [6]. Its mineral phase is primarily composed of hexagonal fluorinated carbonate hydroxyapatite (HAP) crystals. In mature enamel, slender nano-HAP crystals are arranged in parallel to form 3–5 μm diameter rods or prisms, which are considered to be the fundamental organisational units of mature enamel. The rods and inter-rods interweave to form a characteristic ‘fish scale’ structure. The rods extend perpendicular to the enamel surface and forms a 60° angle with the inter-rods [2, 7]. Enamel is formed by ameloblasts and proteins that are in high coordination. Amelogenin (AMEL), which accounts for 90% of the enamel organic matrix, is considered to be the main structural skeleton of amelogenesis. Non-amelogenin is related to the nucleation and growth of crystals, such as enamelin (EMAM) and ameloblasin (AMBN) [8]. It is essential for the special mechanical properties of enamel that a highly ordered crystal structure is formed by precise coordination of ameloblasts and proteins.

**Fig. 1:**
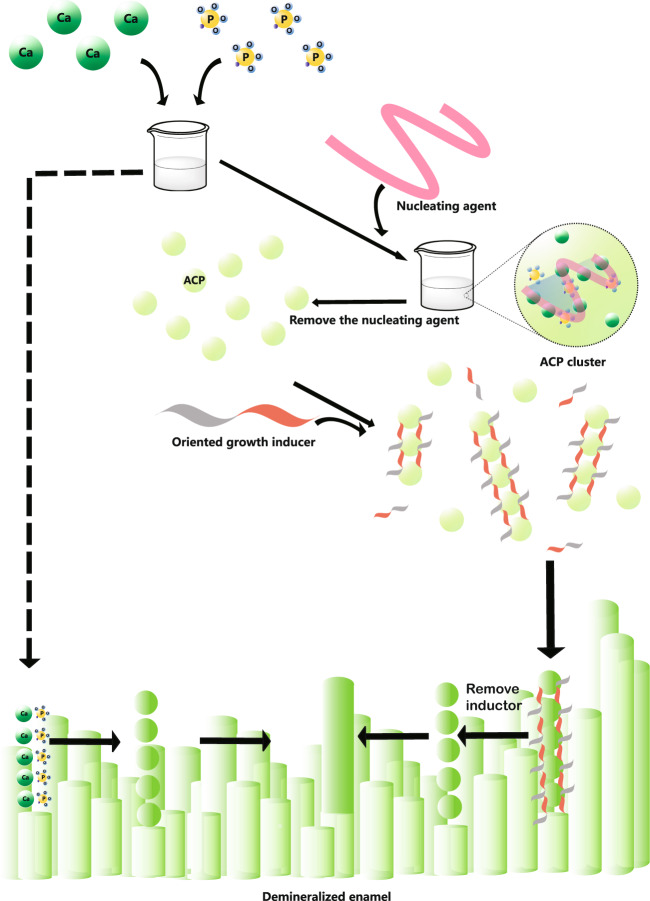
A schematic model of the formation of ACP and transform into enamel-like crystals for biomineralisation. Strategy A (solid arrow): CaP will be stabilised by nucleating agent and form into ACP clusters. After the removement of the agent, the ACP will transform into crystals. Strategy B (dashed arrow): flow of CaP are selected or accelerated into the deep lesions, then nucleate to form ACP and finally transform into HAP

Inspired by amelogenesis, biomimetic systems consisting of the proteins and the proteolytic enzymes involved in amelogenesis have been used in in situ enamel restoration. Since proteins are difficult to manufacture and preserve, biomimetic systems of amorphous calcium phosphate (ACP) stabilised by synthetic materials have been widely studied, including the use of organic matrix with similar functions as the enamel protein matrix and proteolytic enzymes, respectively. To further simplify these systems, flows of calcium phosphate (CaP) are selected or accelerated into the deep lesions, then nucleated to form ACP and finally transformed into HAP (Fig. 1). In this review, after a brief overview of the enamel hierarchical structure and the process of amelogenesis, we detail state-of-art strategies in in situ enamel restoration, as highlighted previously. To summarise the potential problems of in situ enamel remineralization in recent years, we summarised the biomimetic systems into two strategies (Fig. 1), and compared their advantages and disadvantages.

## Mature enamel structure and amelogenesis

Mature enamel consists of carbonated HAP ribbon-like nanowires that are parallel to their *c*-axis. The nanowires have cross-sectional dimensions of ~50 × 25 nm and lengths of up to several millimetres [9]. Approximately 40,000 nanowires at a density of ~550 crystallites/μm^2^ are packed into bundles as rods [10, 11]. At the periphery of the rod bundles, there are ~1–2% organic matrix components, including AMEL, EMAM and AMBN [12, 13]. This well-defined oriented mineralisation pattern is strongly related to the mechanical properties of the enamel, including the hardness and elastic modulus, to ensure a high striking strength [14–16].

The chemical composition of enamel HAP can be partially replaced by different ions; the mechanical properties change accordingly. Sodium (Na^+^), zinc (Zn^2+^) and magnesium (Mg^2+^) ions can be substituted in the calcium position, fluoride (F^−^) and chloride (Cl^−^) ions can be substituted in the hydroxyl position, and carbonate (CO_3_^2−^) ions can be substituted in the hydroxyl and phosphate positions [2, 17, 18]. The hardness of enamel is highly correlated with the zinc content and the presence of zinc in amelogenesis marks the beginning of biomineralisation [17]. Mg^2+^ ions can regulate HAP crystallisation by stabilising ACP because the Mg^2+^ on the surface of the HAP hinders the growth of HAP crystals, thus forming nano-HAP crystals [2, 19, 20]. Fluoride ions increase the acid resistance of enamel [17].

**Fig. 2:**
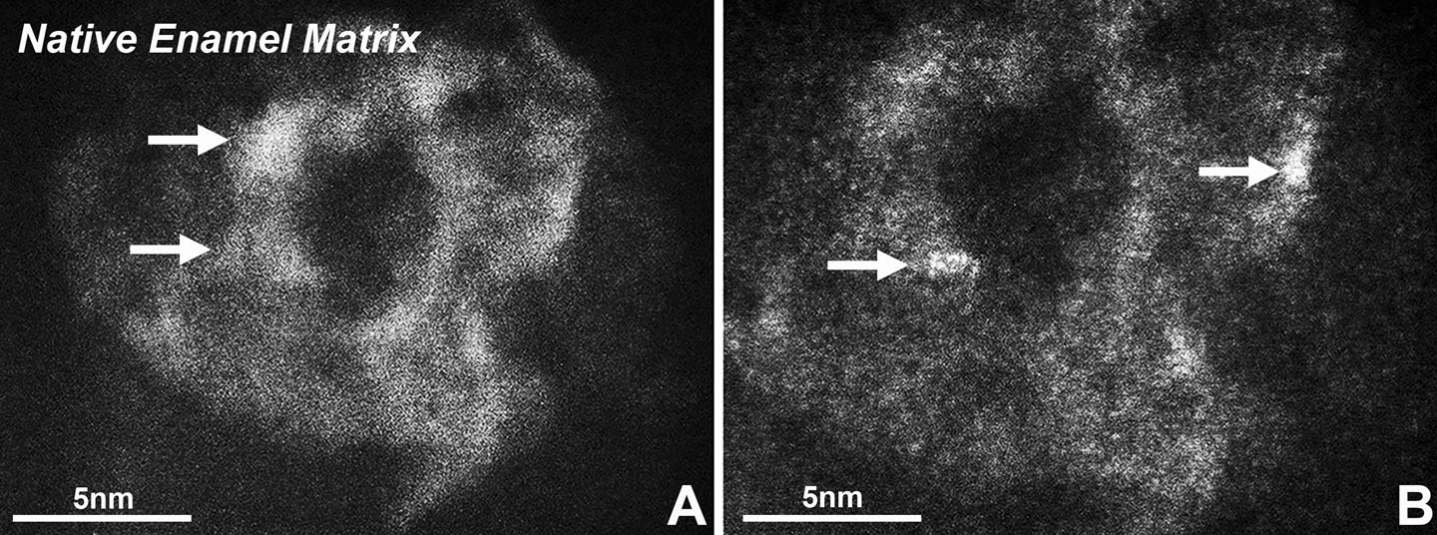
Enamel matrix subunit compartments from native enamel matrix and in ameloblast secretory vesicles imaged using aberration-corrected scanning transmission electron microscopy (STEM) imaging. **A** and **B** are STEM images of different parts of the natural enamel of 1 day postnatal developing mouse molars. Crystal nucleation in the compartment of organic subunits in natural enamel matrix can be seen in both **A** and **B**. Note the individual 1–3 nm diameter nucleation sites (arrows, **A**, **B**) randomly dispersed with a circular 20 nm diameter organic matrix assembly (matrix) (**A**, **B**).

Enamel biomineralisation contains presecretory, secretory, transitional and maturation stages [21]. During the secretory stage, 1–2 nm diameter mineral precipitates aggregate into 5 nm particles in ameloblast secretory vesicles or annular organic matrix subunits, the particles were identified as amorphous calcium phosphate (Fig. 2A, B) [22]. In this stage, ameloblasts extend in the pre-dentin containing vertically oriented collagen fibres and then secrete organic matrix and ACP mineral particles [2, 13, 22–24]. The extracellular organic matrix, which is mainly composed of N-terminal AMEL cleavage products, forms the template of the ACP ribbons, which are assembled by 3–10 particles with diameters of 2–5 nm [2, 22, 25]. The ribbons are parallel to the long axis of the ameloblasts and perpendicular to their distal membranes. After the ribbons reach a thickness of 4–6 µm, the mineralisation front reorganises into rod and inter-rod structures [25].

AMEL, EMAM and AMBN secreted in this stage are secretory calcium-binding phosphoproteins (SCPPs), whose phosphorylation is important in promoting nucleation and stabilising ACP, which is the precursor to HAP crystallisation [12, 13]. Gene defects of the proteins cause amelogenesis imperfecta (AI) in humans [26–29]. In the non-classical theory, SCPPs interact with CaP to form, stabilise and assemble intermediate pre-nucleation clusters, non-crystalline or poorly crystalline mineral phase, in the supersaturated remineralisation solution [16, 21, 30].

AMEL is required by amelogenesis to expand the enamel matrix to accommodate continued enamel ribbon elongation and retrograde ameloblast movement, and to occupy the space between crystals to prevent the fusion of adjacent ribbons in the *a*- and *b*-axis directions; however, AMEL does not directly nucleate, shape or orient enamel ribbons during the secretory stage [13, 22]. This is consistent with the fact that Amelx−/− mice’s enamel ribbons were oriented but fused into fan-like structures [13]. Amelx−/− enamel ribbons do not appear to be crystalline and are composed of ~20 µm thick octacalcium phosphate (OCP); therefore, it seems likely that AMEL interacts with minerals to control the transformation of ACP into HAP and prevent the formation of OCP [13]. During the maturation stage, the ~17 kDa N-terminal AMBN and N-terminal AMEL were identified as residual fragments around the periphery of the enamel rods, which formed an organic network to support the integrity of enamel rod and maintain the hierarchical structure [31].

Matrix metalloproteinase-20 (MMP-20), known as the enamel-specific protease, is co-expressed with SSCPs during the early stage of amelogenenis [32]. SCPPs are hydrolysed by MMP-20 immediately upon secretion into more stable intermediates, which reduces the proteins’ binding sites with CaP [21, 33, 34], followed by the immediate nucleation and transformation of long and thin ribbon-like ACP in the maturation stage [2, 23, 24]. The mutation of the MMP-20 gene causes AI [35]. Kallikrein-4 (KLK-4) completely digests the matrix, which is characteristic of the maturation stage [2, 35]. Hence, the hydrolysis of proteins promotes the transformation of ACP into HAP, which allows the crystallites to primarily grow in thickness and fill the space, resulting in enamel hardening [21, 22, 36]. This is consistent with the discovery of Xie et al. that an increase in protein content is the primary factor that causes the deterioration of the mechanical properties, including stiffness and the elastic modulus [2, 37].

## Biomimetic systems for enamel restoration

As mentioned above, AMEL is essential in amelogenesis because it prevents ACP ribbons from fusing and promotes the directional growth of ribbons [13, 22]. Therefore, AMEL is widely used in hard tissue remineralisation systems. However, based on an in depth understanding of amelogenesis, non-amelogenin also plays an important role in the formation of enamel. Moreover, proteolytic enzymes secreted by ameloblasts, alongside SCPPs, also promote enamel mineralisation by hydrolysing the organic matrix template to affect the mechanical properties [21, 22, 37]. Based on this understanding of in situ enamel remineralisation induced by AMEL in vitro, organic compounds with similar functions as proteins in amelogenesis have been introduced into biomimetic systems.

### Mineralisation induced by proteins

#### AMEL

AMEL contains three important amino acid domains: (1) the hydrophobic tyrosine-rich N-terminal domain, which can bind with other proteins and apatite [21]; (2) the central hydrophobic proline-rich region and (3) the hydrophilic C-terminal domain [36], which can induce the transformation of ACP into ordered crystals [38]. These domains generate the high degree of local supersaturation required for stabilising ACP via interactions with calcium and ensure that the mineral molecules have sufficient time to permeate the deep decay layer [13, 38–40]. AMEL can self-assemble into different structures according to the surrounding in vitro conditions or the hydrolysis of protease, in which the nanosphere is the basis of enamel development [21, 41]. In the nanosphere model, the C-terminal is exposed on the surface and the N-terminal is internally protected [41]. The inner part of the nanosphere stabilises the ACP and arranges it into ribbons, then merges and transforms it into needle-like HAP crystals in parallel arrangements [16].

Despite the surface of the initial carious lesions remaining relatively intact, nano-sized AMEL-CaP clusters can permeate the deep layer through micro-sized diffusion pathways in the inter-crystalline and inter-prismatic space [42, 43]. In situ biomineralisation induced by chitosan (CS)-AMEL was found to result in the formation of a dense interface between the newly grown layer and natural enamel, such that the mechanical properties of the enamel-like layer improved compared to those of etched enamel (Table 1) [44].

**Table 1:** Biomimetic systems based on amelogenin

Biomimetic systems	Characteristics of repair layer					Repair time	Ref.
	Thickness (μm)	Structure of regrown crystals	Orientation degrees I_(002)_/I_(211)_^a^	Modulus (GPa)	Hardness (GPa^1^/VHN^2^)^b^		
Amelogenin-based system							
CS-AMEL	~15	Organized bundles of nanorods	0.95	31.01 ± 8.85	0.98 ± 0.57	3–7 days	[52]
MMP-20-CS-AMEL	–	Organized bundles of nanorods	1.6	1.8-fold CS-AMEL	2.4-fold CS-AMEL^1^	5 days	[33]
2 mg/mL LRAP	–	Organized bundles of nanorods	–	–	197.17 (10.78)^2^	24 h	[50]
0.04 mg / mL LRAP-PPi	~2	Bundles of needle-like crystals	–	–	–	20 h	[49]
0.2 mg/mL LRAP-CS	–	–	1.47	–	1.52-fold demineralized enamel^2^	7 days	[51]
Amelogenin-based peptide system							
P26	~30	Multilayered aprismatic column-liked structure	2.38	1.7-fold demineralized enamel	1.8-fold demineralized enamel^1^	7 days (reapplied on day 3)	[40]
p32	~30	Multilayered aprismatic column-liked structure	1.34	1.8-fold demineralized enamel	1.9-fold demineralized enamel^1^	7 days (reapplied on day 3)	[40]
Peptide A -CMC/ACP	–	Organized bundles of nanorods	1.229 ± 0.094	66.7 ± 2.4	0.70 ± 0.21	7 days	[36]

*CS* chitosan, *AMEL* amelogenin, *MMP-20* matrix metalloproteinase-20, *LRAP* leucine-rich amelogenin peptide, *PPi* inorganic pyrophosphate, *ACP* amorphous calcium phosphate, *TEA* triethylamine

^a^The ratio of diffraction intensity of *c*-axis (002) to another direction (211) has been widely used to evaluate the orientation degree of the apatite crystals [3, 88]

^b^The hardness and modulus of a healthy enamel are measured to be around 4.0 and 90 GPa under the nanoindentation tests, respectively [37]; the Vickers microhardness of healthy enamel is 276–360 VHN [89]

^1^Hardness was tested by nanoindentation test

^2^Hardness was tested by Vickers microhardness test

Leucine-rich amelogenin peptide (LRAP), which is the smallest (with 56–59 amino acids, depending on the species) of the AMEL splice products [45–47], consists of the N- and the C-terminal domains of the full-length AMEL [48]. LRAP can be self-assembled into a nanosphere to form chain-like structure [46]. Non-phosphorylated LRAP preferentially interacts with the *a-* and *b-*surfaces of enamel crystals, inhibiting their oriented growth, to selectively promote linear growth along the *c*-axis of enamel crystals [47–50]. Furthermore, non-phosphorylated LRAP improves the hardness of demineralised enamel (Table 1) [50]. Hence, non-phosphorylated LRAP is often used in enamel biomimetic mineralisation [47, 49, 50]. Phosphorylated LRAP stabilises ACP [49]. When phosphorylated LRAP and CS were applied to the demineralisation of enamel for 3 days, the newly formed crystal was similar to the crystal formed in AMEL-CS but the rate of nucleation and growth of HAP in LRAP-CS was faster [47, 51]. The hydrophilic nature of LRAP may explain this due to the presence of highly charged residues that promote the recruitment of Ca^2+^ and PO_4_^3^^−^ ions to the apatite surface. Furthermore, the residues promote interaction with the apatite surface, resulting in CaP precipitation. LRAP has a time-dependent regulation of crystal growth and morphology. From day 3 to day 7, the orientation of the crystals gradually improved in the above-mentioned study. After 1 week, the orientation of the crystal did not significantly improve. Despite the relatively uniform crystal size, the crystal was partially melted and the density increased, forming densely packed rod-like crystals (Table 1) [51].

**Fig. 3:**
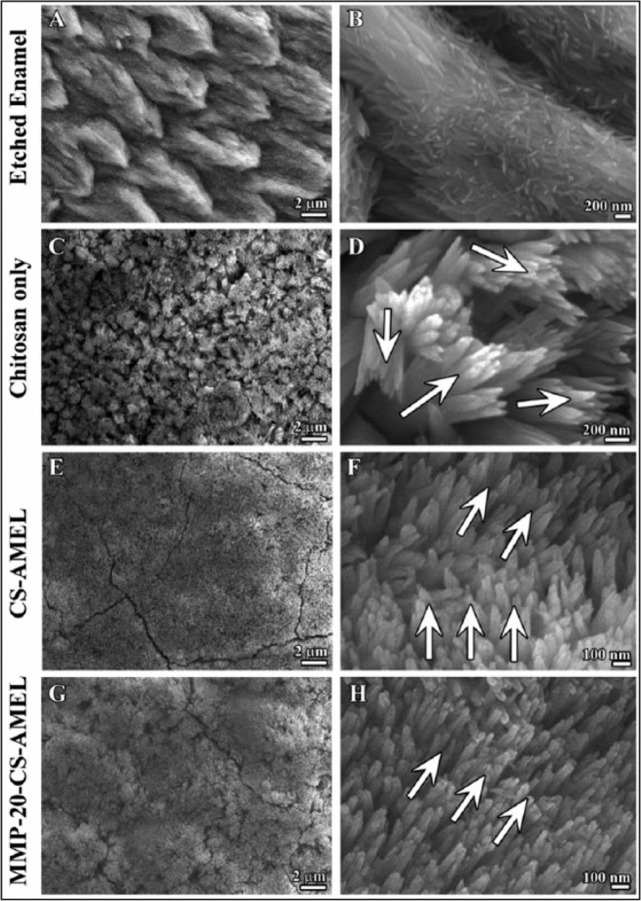
Microstructure of enamel-like apatite crystals in the newly grown layer. Scanning electron microscopy (SEM) images showing **A, B** etched enamel, newly grown hydroxyapatite crystals in CS (**C, D**), AMEL-CS hydrogel (**E, F**) and AMEL-CS-MMP-20 (**G, H**). Arrows in **D, H** and **F** indicate the crystal orientation [33]

However, the mechanical properties of the newly grown minerals were not as good as those of natural enamel when AMEL and LRAP were applied into in situ enamel restoration [42, 44], because of the lack of an extremely fine structure caused by protein occlusion [52]. Protease prevents protein occlusion inside apatite crystals and allows apatite crystallites to primarily grow in thickness and fill the space, resulting in the enamel hardening [21, 22, 33, 35, 36]. AMEL and other enamel matrix proteins are hydrolysed by MMP-20 at the beginning of secretion and finally removed by KLK-4 at the maturation stage [33]. Hence, the effect of protein occlusion can be eliminated by adding protease into biomimetic systems [33, 35, 53]. When MMP-20 was added into CS-AMEL for in situ remineralisation, AMEL was gradually degraded by MMP-20. The newly formed crystals were more uniform, oriented and crystallised than those without MMP-20, and their mechanical properties were closer to those of natural enamel (Table 1) (Fig. 3) [33]. The process of adding AMEL and MMP-20 to CS imitates the process by which ameloblasts simultaneously secrete both AMEL and MMP-20 during the secretion stage. Furthermore, the combination of AMEL and MMP-20 has made substantial progress towards the goal of in situ enamel restoration. Nevertheless, the synthesis process of AMEL and MMP-20 is complex and expensive, and the soft intermediate materials produced in the synthesis process cannot bear the masticatory pressure on the occlusal surfaces during mastication. To further imitate the physiological process of amelogenesis, other enamel matrix proteins should also be added into the biomimetic system.

#### Non-amelogenin

AMEL belongs to the class of intrinsically disordered, or unstructured, proteins, which lack a regular secondary or tertiary structure. Nevertheless, AMEL can transform into a folded state following interactions with its targets (such as non-amelogenin or apatite) and as part of its overall function [21].

One of the targets of AMEL is AMBN. AMBN is the second most abundant protein in the extracellular matrix of enamel, which is rich in proline/glutamine [54], and may interact with AMEL via the lectin-like binding domain of AMEL [55]. Non-phosphorylated AMBN has low solubility and can combine with Ca^2+^. Phosphorylated AMBN is a soluble signal molecule during the development of enamel [56]. The ~17 kDa N-terminal of AMBN and N-terminal of AMEL colocalise around the periphery of the enamel rods. The association of these residual protein fragments creates a discontinuity between the enamel rods, which is essential for supporting the integrity of enamel rods and maintaining the hierarchical structure of the enamel [31]. The C-terminal of AMBM is glycosylated and sulfated and has a relatively high affinity to Ca^2+^ [57].

ENAM, the least present protein (3–5%) in enamel, is also one of the AMEL targets and is a type of macromolecular glycoprotein [12, 58, 59]. The surface of ENAM-mutated hypoplastic enamel appears to be normal. However, severe sub-nano and micro-structural defects appear beneath the subsurface layer [60]. The ~32 kDa cleavage product of ENAM is a functional fragment. Its N-terminal has a phosphorylated serine, which is the binding site for AMEL and minerals [12, 58, 59]. The N-terminal is located in the newly formed enamel matrix, while the C-terminal is located in the more mature enamel matrix [12]. AMEL alone was a weak nucleation promoter but the addition of ENAM-promoted nucleation in a highly nonlinear, nonmonotonic manner, reaching a sharp maximum at a ratio of 1:50 ENAM/AMEL [58]. It is possible that only isolated ENAM can enhance nucleation, while ENAM oligomers cannot [58]. The excess or lack of ENAM affects the formation of rods since isolated ENAM increases rapidly with increasing ratio, resulting in the increasing nucleation rate. However, when the ratio exceeds 1:50, ENAM exists in the form of an oligomer, which reduces the nucleation promoting sites such that the nucleation rate decreases [58].

However, there are few studies on the application of non-amelogenin in the biomimetic mineralisation of enamel at present. It is necessary to further study its synergistic effect with AMEL on mineralisation. It will be beneficial to develop and perfect biomimetic enamel mineralisation materials to restore the ordered oriented mineralisation of enamel.

#### Amelogenin-based peptide

Since the synthesis of full-length amelogenin is time-consuming and expensive, the biomimetic mineralisation of enamel under physiological conditions can be realised by imitating the self-assembling structure of AMEL and retaining its functional domains [36, 38, 40]. In addition, the synthesis of new synthetic peptides by permutation and combination of the functional fragments of AMEL can enhance the understanding of the role of the functional fragments of proteins in enamel formation.

Ame-CT16 (LEAWPATDKTKREEVD) and HA6-1 (SVSVGMKPSPRR) are both C-terminal domains of AMEL. Ame-CT16 guides ACP to form ordered, oriented crystals and HA6-1 guides ACP nanoparticles to specifically bind to the enamel surface [36]. The SVSVGMKPSPRR-GGGGS-LEAWPATDKTKREEVD sequence, named peptide A, was formed by binding the two peptides with a variable linker peptide (GGGGS) to induce in situ mineralisation, which promoted the orientation of ACP nanoparticles to form enamel-like ordered crystals with high mechanical strength and caused the newly formed mineralised layer to closely adhere to the enamel surface [36].

**Fig. 4:**
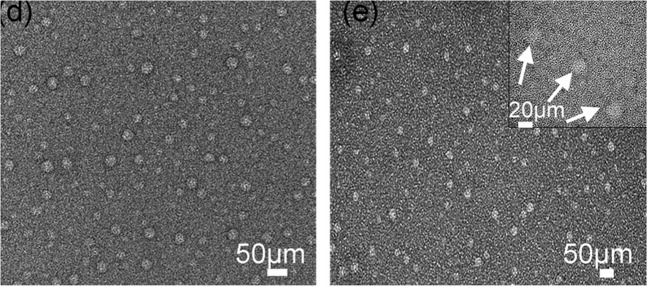
Transmission electron microscopy (TEM) images of nanospheres formed from peptides P26 (**d**) and P32 (**e**) at pH 7.4 at 25 °C. Reproduced with permission from ref. [40]

**Fig. 5:**
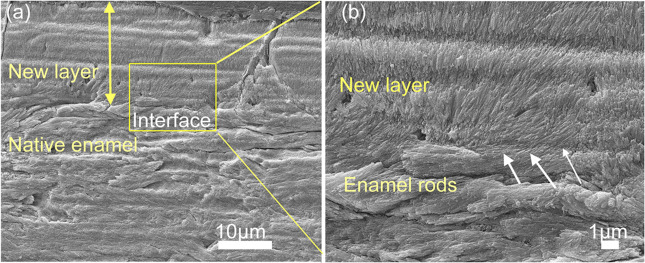
SEM images of the restored HAP layers treated in P26 after 7 days of incubation in artificial saliva in pH 7.0 at 37 °C. **a** Cross-sectional view of regenerated HAP layers. **b** Magnified image of (**a**) (yellow square) depicts the newly formed perpendicularly stacked crystals with a seamless attachment interface with underlying enamel rods. Reproduced with permission from ref. [40]

Two synthetic AMEL-inspired peptides of 26 and 32 amino acid residues (P26 and P32, respectively) remain the last 12 amino acid residues at the C-terminal of AMEL (with the ability to bind to HAP, which promotes crystal nucleation and preferred orientation crystallisation) and 14 amino acid residues remain at the N-terminal of AMEL (including phosphorylated serine, which adjusts the crystal shape and stabilises ACP). P32 also has two proline repeats in the middle segment of AMEL. The synthesised peptides can spontaneously assemble into nanospheres (Fig. 4) and form multilayer aprismatic enamel on the demineralised enamel (Table 1) (Fig. 5). The elastic modulus and hardness of HAP are twice that of demineralised enamel and the HAP is closely combined with the underlying enamel. This process is dose-dependent [38]. The restorative layer lacks a columnar structure and the dense crystals are arranged in parallel, which can resist acid penetration. That is to say, P26 and P32 promote crystal nucleation, improve the preferred orientation of the crystal and reduce the size of crystal. The difference in crystal size between the restored enamel induced by P26 and P32 was not statistically significant; however, more proline repeats may lead to significant changes in crystal size [40]. However, the inverse relationship between crystal size and hardness is still worth studying. Therefore, it will be helpful to improve the design of biomaterials to continue exploring the domains that control peptide assembly, adjust the crystal size and improve crystal orientation [40].

### Mineralisation induced by ACP

In general, apatite crystals nucleate as ACP [61, 62]. At the beginning of mineralisation, CaP ion clusters, which are ACP precursors, result in the nucleation of ACP. Nascent CaP precipitates are dual amorphous phases that transition between individual phases through dehydration [61, 62]. Further transformation into crystalline apatite occurs either through internal lattice rearrangements or reprecipitation of constantly dissipating units in contact with an aqueous solution [62]. According to the above non-classical theory, ACP is widely used for in situ enamel remineralisation. For example, casein phosphate polypeptide (CPP)-ACP has been added into toothpaste to achieve commercialisation. CPP is a bioactive agent derived from dairy products, which can stabilise supersaturated CaP ions in an oral environment. When the pH decreases, stable ions are released; phosphorus ions buffer the pH condition and Ca^2+^ promotes remineralisation [63]. The remineralisation ability of the system has been proven by in vitro and in vivo studies [64, 65]. The combination of toothpaste or MI Varnish containing CPP-ACP and F^−^ improves the remineralisation effect of enamel after 3 months [63, 66]. In addition to CPP-ACP, CS and triethylamine (TEA) are also used to stabilise ACP and promote in situ enamel remineralisation.

#### CS

CS is a linear chain polysaccharide that contains β-(1–4)-linked D-glucosamine (as the deacetylated unit) and N-acetyl-D-glucosamine (as the acetylated unit). Rich amino groups enable CS to adjust its physical and chemical properties according to the pH value of the environment [52]. When the pH value is below the pKa of CS (6.5), CS can capture H^+^ to become soluble and release stabilised ions similar to CPP-ACP, as mentioned in the previous section [63]. Simultaneously, CS interacts with the negatively charged etching enamel surface, which is mainly caused by the mucin adsorbed on the enamel surface, resulting in the formation of a barrier to prevent the enamel surface from further etching [67, 68]. Furthermore, CS can carry proteins to prevent it from losing into saliva through electrostatic interaction [42, 52]. When the pH increases, the electrostatic effect of CS weakens and the proteins loaded on CS are released [42, 52]. The microgel formed with CS and CaP particles that are larger than 100 nm has an induction stage during the initial application. During this period, ACP and poorly crystalline CaPs start to become re-dissolved, and CaP is re-deposited on the surface of the etched enamel. Seven days after the application of the microgel, the rods and inter-rods gradually form grain-like minerals with a diameter of ~50 nm (Table 2) [69]. Bacterial biofilms tend to gather on the rough surface. If biomineralisation materials cannot inhibit the colonisation of bacteria in the mouth, the formation of dental plaque and dental calculus may be promoted. In recent years, carboxymethyl chitosan (CMC), which is a derivative of CS containing a carboxymethyl group, has also been used in biomineralisation studies [16, 36, 70, 71]. CMC can inhibit cariogenic bacterial adherence, co-adhesion and biofilm formation on the enamel surface, possibly by altering the bacterial surface charge and enhancing the flocculation effect. Furthermore, 1% CMC has no effect on the survival and growth of cariogenic bacteria after 24 h; therefore, it will not damage the balance of normal oral flora [71]. To summarise, CS and its derivative have the following functions: (1) the protection of the enamel from acid etching; (2) acting as ideal protein carriers in an oral environment with dynamic pH changes; (3) the provision of necessary ions for remineralisation, as a reservoir of mineralised ions and (4) the prevention of early caries.

**Table 2:** Biomimetic systems based on ACP

Biomimetic systems	Characteristics of repair layer					Repair time	Ref.
	Thickness (μm)	Structure of regrown crystals	Orientation degrees I_(002)_/I_(211)_^a^	Modulus (GPa)	Hardness (GPa)^b^		
Chitosan system							
CS	–	–	0.5	–	–	5 days	[33]
CS-HAP microgels	–	Grain-like mineral	–	–	–	7 days	[69]
CMC-ACP-peptide A	–	Oriented enamel-like crystals	1.491 ± 0.032	43.5 ± 1.7	0.48 ± 0.10	7 days	[36]
CMC-ALN/ACP-Gly	–	Oriented and ordered needle-like crystals	–	–	–	7 days	[16]
TEA	~2.7	Organized enamel rods and inter-rods	–	87.26 ± 3.73	3.84 ± 0.20	2 days	[7]

*CS* chitosan, *HAP* hydroxyapatite, *CMC* hydroxymethyl chitosan, *ACP* amorphous calcium phosphate, *Gly* glycine, *TEA* triethylamine

^a^The ratio of diffraction intensity of *c*-axis (002) to another direction (211) has been widely used to evaluate the orientation degree of the apatite crystals [3, 88]

^b^The hardness and modulus of a healthy enamel are measured to be around 4.0 and 90 GPa under the nanoindentation tests, respectively [37]

**Fig. 6:**
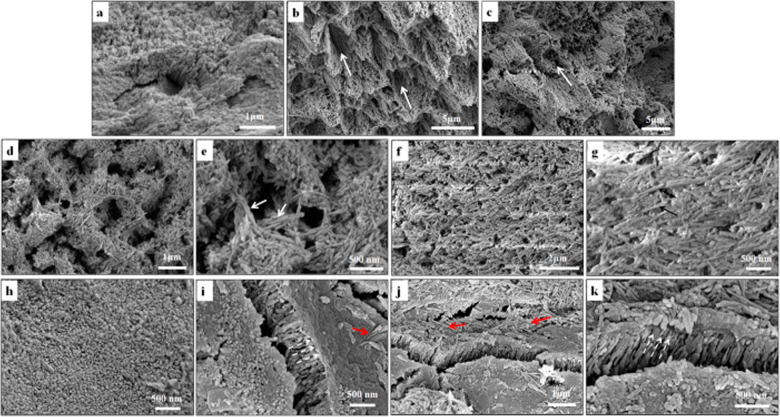
Restoration of the complicated structure of enamel by CMC-ALN/ACP-Gly. SEM results showing the surface morphology of normal enamel in (**a**). The surface morphologies of demineralized enamel (**b, c**); remineralised enamel with CMC/ACP nanocomplexes (**d, e**); remineralised enamel with CMC-ALN/ACP nanocomplexes (**f, g**); remineralised enamel with combination of CMC-ALN/ACP nanocomplexes and Gly (**h–k**), showing layers of oriented and ordered mineral crystals perpendicular to the enamel surface indicated with arrows of white dash line, meanwhile, on these layers scattered mineral crystals parallel on the enamel surface indicated with arrows of red dash line. Reproduced with permission from ref. [16]

However, CS has the following problems. From Table 2, CS itself cannot induce ACP to form an orderly linear chain structure, cannot specifically combine with the enamel surface, and induces a mineralisation degree of the restored layer that is lower than that of natural enamel [16, 36]. To improve the affinity of CS to the enamel surface, studies have conjugated alendronate (ALN) with the carboxyl-terminated poly (amido amine) of CMC. Two phosphate groups of ALN can replace two phosphate groups on the HAP surface to bind with HAP [16]. However, the ACP stabilised by CMC-ALN alone has difficulty transforming into HAP for the residual organics in the newly grown layer, which can lead to low mechanical properties [36, 52]. Sodium hypochlorite (NaClO) has been added to degrade CS by attacking the β-(1,4) glucoside bonds, similar to the protease that decomposes amelogenin in vivo. With a small amount of NaClO, nano-ACP rapidly transforms into HAP. Then, glycine (Gly) or peptide A, as mentioned previously, is added to guide the HAP/ACP nanoparticles to organise into well-ordered rod-like apatite crystals (Fig. 6) [16, 36]. This process is very similar to amelogenesis and conforms to the non-classical theory. It provides great hope for the in situ biomimetic restoration of enamel. However, the use of toxic chemicals (hypochlorite and bisphosphonates (BPs)) and soft intermediate materials produced in the synthesis process may limit its clinical use.

In addition to NaClO, lysozyme, which exists in oral saliva, also degrades CS into glucosamine by cleaving β-(1,4) glucoside bonds. The incorporation of lysozyme to CS hydrogels accelerates the degradation rate of CS in a dose-dependent manner [72]. Lysozyme has a phosphorylated serine and rich carboxyl group, which can also chelate Ca^2+^ and promote nucleation through electrostatic interactions [59]. As a result, lysozyme is a better substitute than NaClO for degrading CS during biomimetic enamel restoration. However, it is unknown whether the CS products after the cleavage of NaClO or lysozyme will affect the mechanical properties of the newly grown layer. Furthermore, the lysozyme outside the biomimetic system (such as in saliva) may disturb the electrostatic interactions between CS and its targets. Therefore, the effect of saliva on the efficacy of CS must be evaluated [42].

#### TEA

**Fig. 7:**
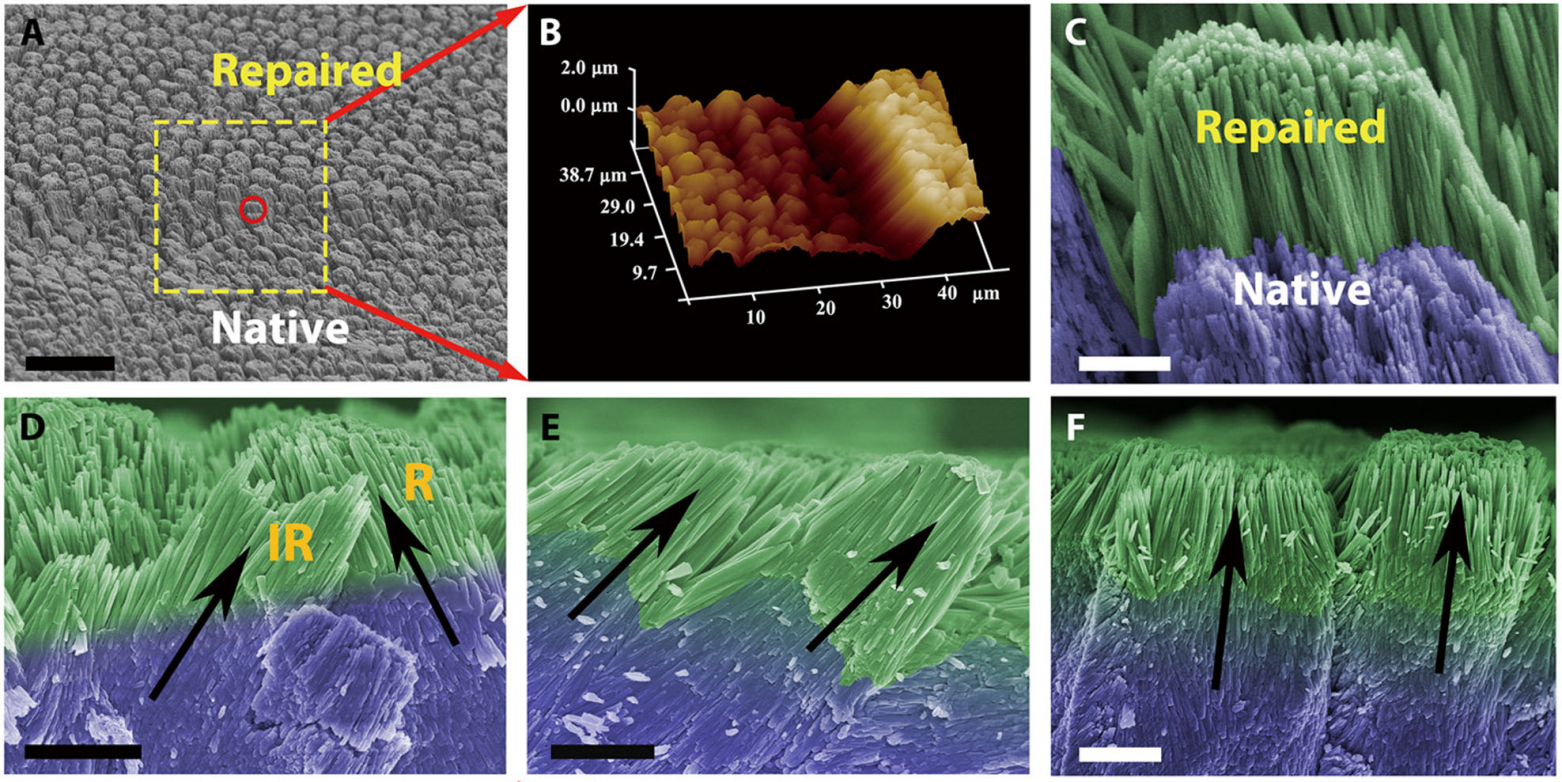
Restoration of the complicated structure of enamel by TEA. **A** SEM image showing both etched enamel and repaired enamel. **B** A three-dimensional atomic force microscopy (AFM) image of repaired enamel. **C** High-magnification SEM image of the red circle in (**A**). **D** Cross-sectional view of final repaired enamel, where both enamel rods and inter-rods were repaired. Rods and IR represent for enamel rod and inter-rod, respectively. **E, F** Enamel rods with different orientations can be repaired [7]

A small amount of the organic small molecule TEA can be used in ethanol as a stabiliser to form stable CaP ion clusters [7, 70]. An ethanol solution was dropped onto a demineralised enamel surface and air-dried at 25 °C for 5 min. With the gradual volatilisation of TEA, the CaP ion clusters formed a continuous ACP precursor layer on the HAP of natural enamel without any gaps. Subsequently, the HAP crystals extended along the *c*-axis. Simultaneously, the enamel rods and the inter-rods grew epitaxially. The hierarchical structure of the restored enamel was the same as that of natural enamel and even slightly improved the mechanical properties (i.e. the carbonate content of the repaired HAP was lower than that of natural HAP) (Fig. 7) [7]. Since TEA volatilises entirely with ethanol at room temperature, it does not remain in the newly formed mineralised layer. Furthermore, the mechanical properties do not reduce, dissimilar to the restored enamel induced by proteins or CS (as shown by the modulus and hardness values in Tables 1 and 2). Therefore, it is unnecessary to add protease and other substances to decompose the organic matter, which greatly simplifies the process of remineralisation and greatly reduces the cost for future extensive applications of in situ enamel remineralisation systems. However, due to the poor stability of the ACP that is formed in the system, there is a limit for the remineralisation thickness (~2.8 μm). This defect can be remedied by enhancing the stability of ACP or reusing the biomimetic system.

### Mineralisation induced by ions flow

**Fig. 8:**
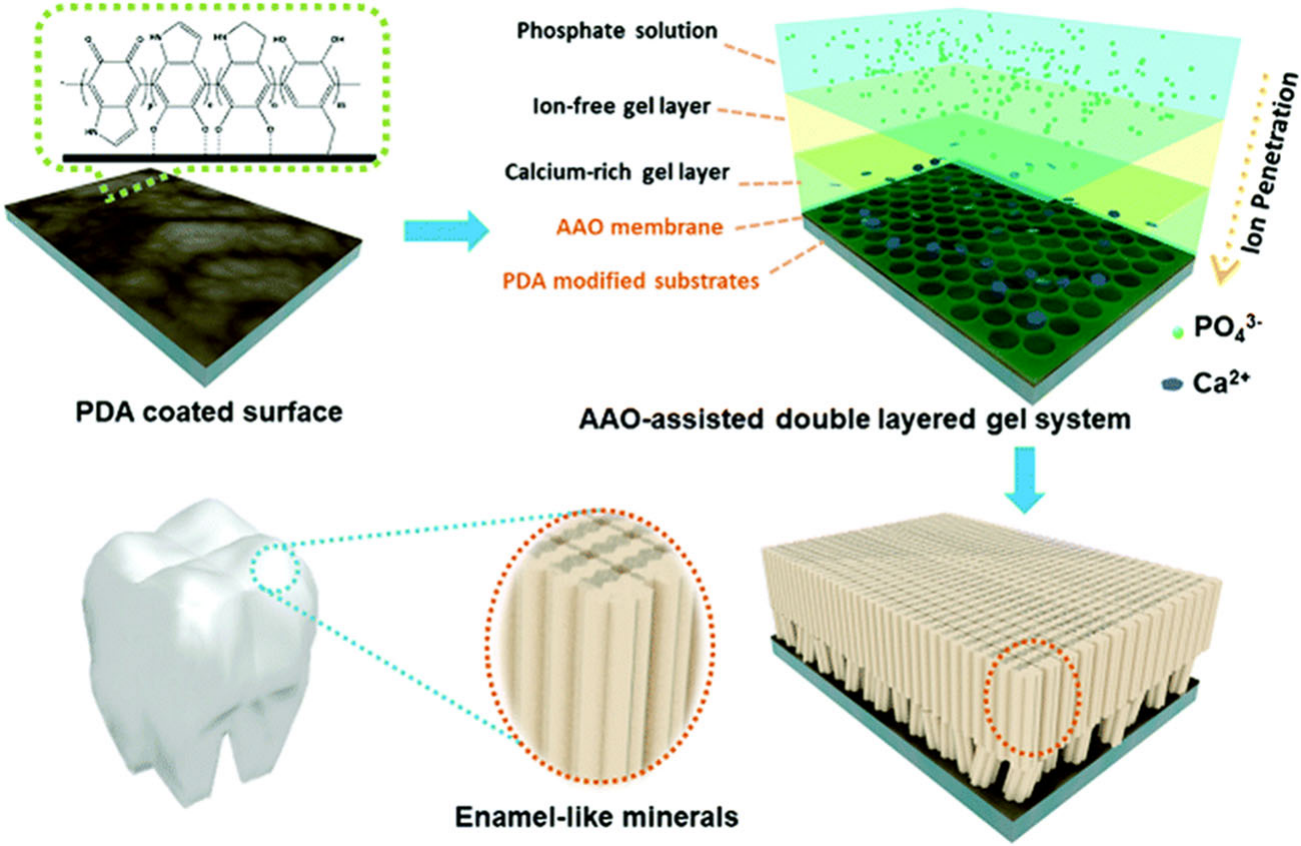
Schematic of the ADGS. The PDA-modified substrates were coated by AAO membrane, a calcium-containing gelatin hydrogel and an ion-free gelatin hydrogel, from bottom to top. Phosphate solution was added to the top for mineralisation through ion diffusion. Reproduced with permission from ref. [73]

Highly oriented HAP crystallites were produced by a biomimetic anodic alumina oxide (AAO)-assisted double-layered gel system (ADGS), exhibiting an elastic modulus of 52.14 + 8.48 GPa and a nanohardness of 0.73 + 0.23 GPa, which are close to the values of natural enamel [73]. The system is not only suitable for the demineralised enamel surface but also can initiate the formation of enamel-like minerals on the surface of other substrates (such as titanium and silicon plates). In this system, polydopamine (PDA) modified the substrates and carried AAO membranes, a calcium-containing gelatin hydrogel, and an ion-free gelatin hydrogel in sequence from bottom to top. Then, the phosphate solution was added to the top for mineralisation through ion diffusion (Fig. 8) [73]. PDA, as a nucleator, chelates with Ca^2+^ through the phenol hydroxyl group and causes the crystals formed after mineralisation to closely combine with the surface of the substrates. The AAO membrane guides the inflow of ions and the capillary action in the membrane causes ion transport, which accelerates ion transport and produces more nucleation sites [73]. The selective flow of ions mimics the early stage of enamel formation [74]. Similar to the hydrogel and proteins mentioned in previous sections, gelatin was introduced into the system as both a template and ion transportation medium in which the minerals could grow [75, 76]. The introduction of gelatin also made it possible to stay in the formed minerals, so that its mechanical properties remained lower than those of natural enamel. Furthermore, this kind of soft material cannot bear the masticatory pressure on the occlusal surfaces during mastication.

**Fig. 9:**
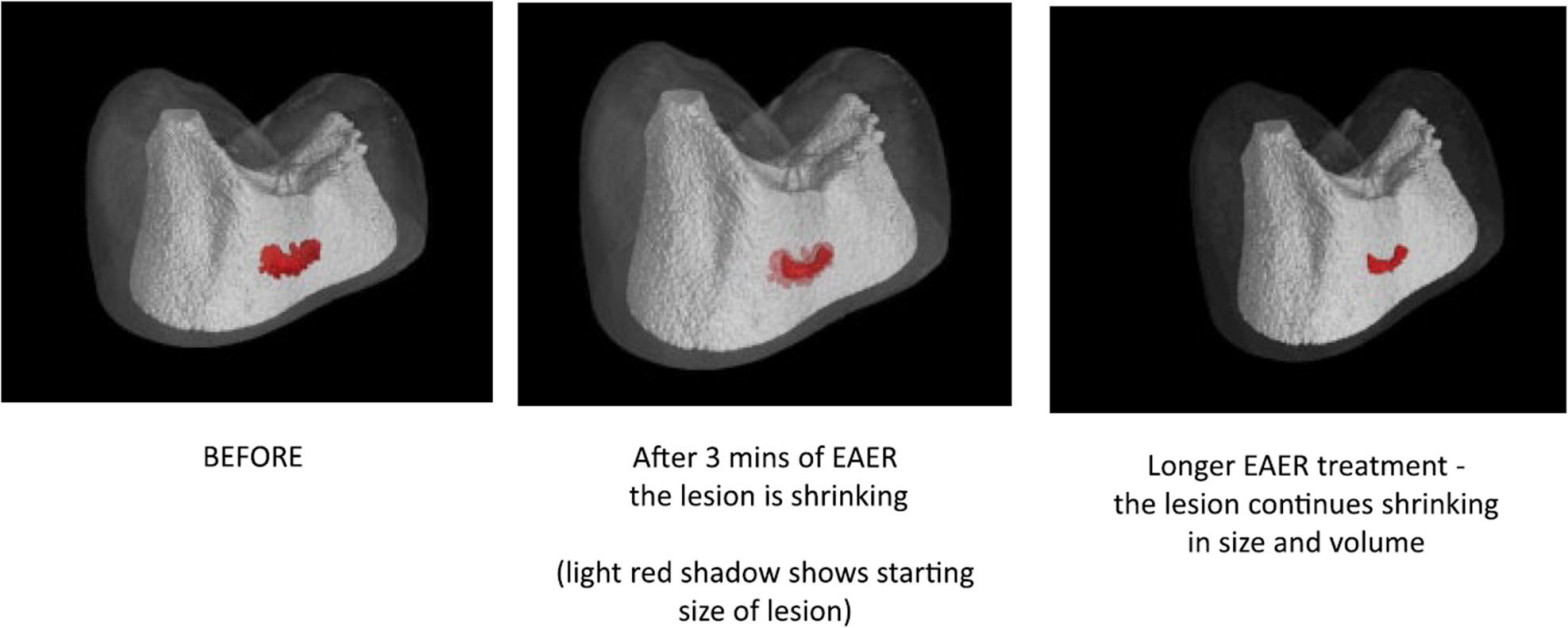
Three-dimensional X-ray mercury cadmium telluride images of an EAER-treated lesion. EAER-treated lesion reducing in volume and size. The red colouring represents the area of the enamel lesion that had a calculated mineral density ≥5% lower than the surrounding ‘healthy’ enamel [82]

**Fig. 10:**
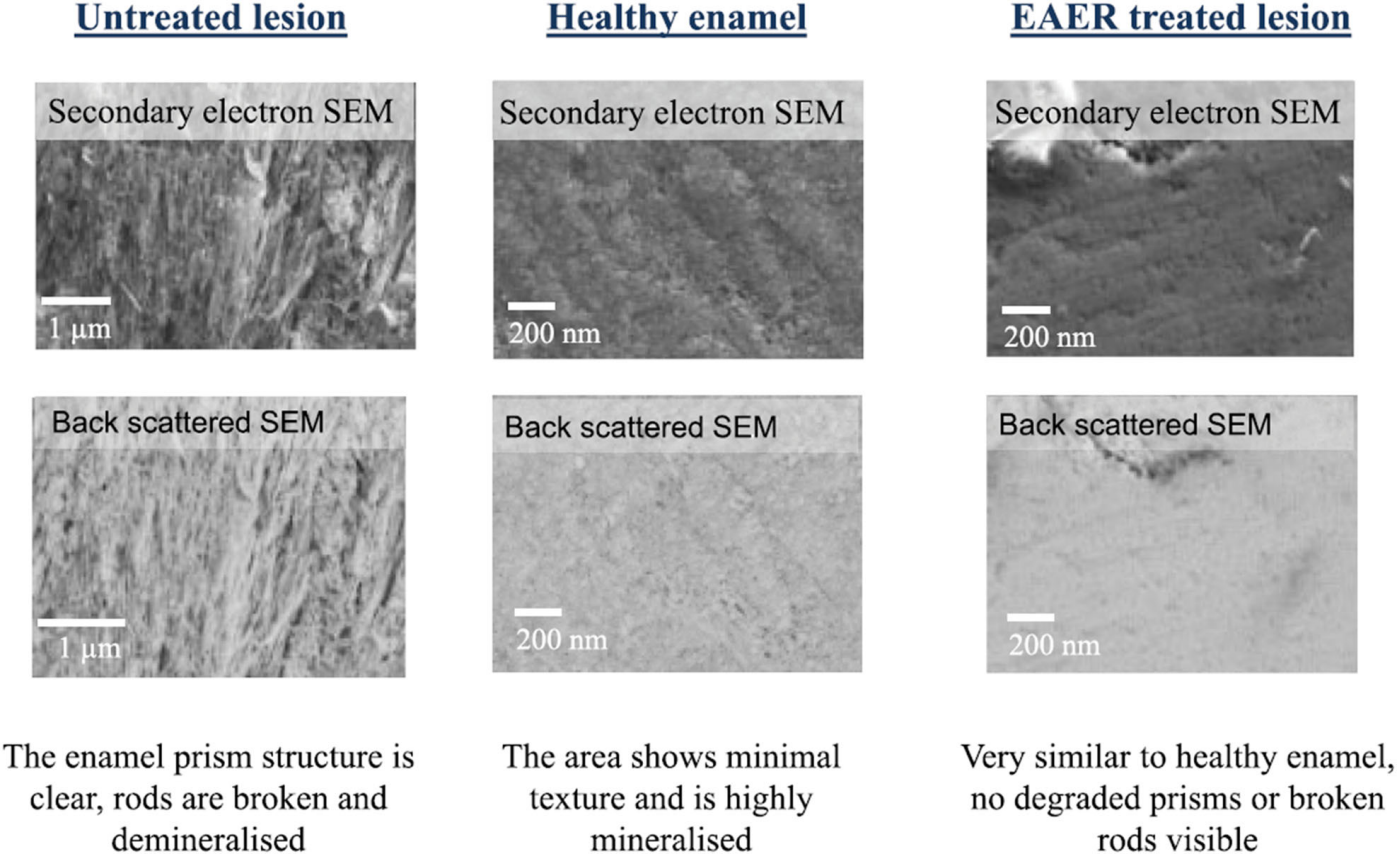
SEM examinations of demineralised enamel and EAER-treated enamel. In the untreated lesions, the enamel rod structure is clear: rods are broken and demineralized. However, enamel in the EAER-treated lesions is very similar to nature enamel, with no degraded rods or broken rods visible [82]

Electrically accelerated ions flow via iontophoresis was used in the treatment of dentin hypersensitivity as early as the 1960s [77]. Iontophoresis is beneficial for accelerating and enhancing the remineralisation of enamel after etching [78, 79]. Compared with diffusion alone, electrically accelerated ions flow delivers CaP ions into enamel sections to significantly greater depths (~1 mm) and does not require drilling holes for the treatment of initial-stage and moderate caries lesions [77, 80–82] Reinomiva, a King’s College London dental spinout company, formed a commercialised method that utilises iontophoresis, known as electrically accelerated and enhanced remineralisation (EAER) [82]. The basic steps of EAER are as follows. After cleaning the surface of the lesion, a paste or liquid remineraliser is placed on the lesion to form a mineral reservoir. An electrode is then placed in the remineraliser and an opposite electrode is placed in a suitable location (e.g. on the gingival surface) to create an electric field. After electrifying, the remineraliser is accelerated towards the opposite electrode, which does not damage the surrounding tissues [80, 82]. Through noninvasive micro-computerised tomography, it can be clearly observed that the lesions of tooth demineralisation samples using EAER are significantly smaller in depth and volume than those in the diffusion group (Fig. 9) [82]. Since there is no need for an additional organic matrix in the use of EAER, the enamel treated by EAER is harder than that of natural enamel. Unlike proteins and CS, EAER cannot capture CaP or form an organic template. However, the appearance of EAER-treated lesions is very similar to that of natural enamel, and there no broken rods or deteriorated prisms have been observed under scanning electron microscope (Fig. 10) [82].

### Mineralisation induced by BPs

BPs, which are pyrophosphate analogues, are a class of drugs used in various bone diseases and metabolic diseases, that specifically combine with HAP. The oxygen atom that connects the two phosphonic acid groups in inorganic pyrophosphate (PPi) is replaced by a carbon atom in BPs, forming a more stable P–C–P structure. PPi mainly inhibits crystal growth by combining with Ca^2+^ [38, 49]. The inhibition of CaP crystal growth has been attributed to the adsorption of PPi to the HAP crystal growth sites [83]. The concentration of PPi is reduced by surface-catalytic hydrolysis of the enamel surface to reverse its mineralisation inhibition, resulting in the preferential formation of minerals on the acid-etched enamel surface. However, PPi cannot induce oriented mineralisation. The application of PPi alone leads to the formation of randomly distributed large non-adherent plate-like HAP crystals. Therefore, PPi must be combined with other oriented mineralisation-inducing materials (like LRAP) to control the start and rate of biomimetic enamel restoration [49]. Polyethylene glycol (PEG) functionalised with a PPi tooth-binding anchor could inhibit the adsorption of salivary protein to the tooth surface after rapid binding to the surface. The increase in PPi content could promote the binding ability to the enamel surface. This may be because protein binding sites on the HAP surface are occupied by PPi, or because PPi-PEG forms a neutral hydrophilic layer on the HAP surface, which effectively reduces the hydrophobic interaction between the salivary protein and bacteria on the HAP surface [84]. Therefore, BPs as PPi analogues may have similar effects.

Unlike PPi, BPs have more stable physical and chemical properties and have resistance to multiple enzymes in the human body. The ability of BPs to resist bone resorption increases with the extension of the side chain. The side chain structure with four carbon atoms as the skeleton has the strongest activity, i.e. ALN. ALN interacts with the HAP surface through ligand exchange, in which two phosphate groups of the ALN molecule replace two surface phosphate groups of HAP [85]. Therefore, ALN has been coupled with different drug carriers to form bone- or tooth-targeted drug delivery systems [85, 86]. ALN has been conjugated on carboxyl-terminated poly(amido amine) (PAMAM-COOH) with remineralisation potential to increase the bonding strength between PAMAM-COOH and the enamel surface [86]. Furthermore, CMC has been conjugated with ALN to stabilise ACP nanoparticles to form CMC/ACP, which can specifically adsorb on the enamel surface. Guided by glycine, the remineralisation system can form oriented and ordered biomimetic remineralisation on acid-etched enamel surfaces [16].

## Conclusions

Caries is a dynamic disease that is caused by the disequilibrium of demineralisation and remineralisation [36, 52]. If the demineralisation rate is greater than the remineralisation rate, then minerals gradually lose and eventually form irreversible damage and caries cavities [52]. If the remineralisation is dominant, the minerals gradually deposit and the caries can be repaired. Therefore, promoting remineralisation in the early stage of caries can achieve the goal of noninvasive treatment, which is of considerable clinical significance. So far, fluoride treatment is still the most widely used remineralisation method for early enamel caries. However, fluoride has its limitations; the addition of fluoride can only accelerate remineralisation in the initial stages. In the subsequent stages, the process tends to be stable, even under the action of a high concentration of fluoride. Moreover, the systemic application of fluoride has a limited effect on caries prevention. Therefore, it is still necessary to find alternative methods [87].

One of the current strategies of in situ biomimetic mineralisation of enamel is to select and manufacture organic materials with lower costs and the ability to stabilise ACP and promote oriented crystallisation, like AMEL and non-amelogenin (Fig. 1). In addition, proteolytic enzymes or analogues are added to hydrolyse organic materials to prevent organic materials from being embedded in crystallines and affecting their mechanical properties. However, the effects of non-amelogenin on biomimetic enamel mineralisation should be considered in future experiments; whether proteins in saliva, such as lysozyme, proline-rich proteins and casein-rich proteins, affect the efficacy of biomimetic mineralisation guided by AMEL should also be evaluated [21, 42]. Due to the limited ability of materials for stabilising CaP clusters, the poor stability of ACP and the long time required for ACP to enter the biomimetic mineralisation front, ACP transforms into the more stable HAP before reaching the biomimetic mineralisation front. Therefore, another strategy was born. Through either unidirectional selection or an accelerated ion current into the depth of demineralised enamel, CaP ion clusters can form ACP in enamel (Fig. 1). Both strategies aim to avoid the mechanical property degradation caused by the accumulation of the organic matrix among apatite crystals. This approach is correct. In all systems, TEA and EAER-guided remineralisation had no retention of the organic matrix and their mechanical properties recovered best after remineralisation. However, these systems have a lack of in vivo evidence. It is still unknown whether many factors in the body will affect their remineralisation effects. However, these techniques highlight significant progress towards in situ enamel biomimetic remineralisation technology.

## Abbreviation

HAP: hydroxyapatite
AMEL: amelogenin
EMAM: enamelin
AMBN: ameloblasin
ACP: amorphous calcium phosphate
CaP: calcium phosphate
SCPPs: secretory calcium-binding phosphoproteins
AI: amelogenesis imperfect
OCP: octacalcium phosphate
MMP-20: Matrix metalloproteinase-20
KLK-4: Kallikrein-4
CS: chitosan
LARP: leucine-rich amelogenin peptide
IDPs: intrinsically disordered, or unstructured, proteins
CPP: casein phosphate polypeptide
TEA: triethylamine
CMC: carboxymethyl chitosan
ALN: alendronate
NaClO: sodium hypochlorite
Gly: glycine
AAO: anodic alumina oxide
ADGS: anodic alumina oxide-assisted double-layered gel system
PDA: polydopamine
EAER: electrically accelerated and enhanced remineralization
BPs: biophosphonates
PPi: inorganic pyrophosphate
PEG: polyethylene glycol
PAMAM-COOH: carboxyl-terminated poly(amido amine)
